# The experience of stigma in family caregivers of people with schizophrenia spectrum disorders: A meta-synthesis study

**DOI:** 10.1016/j.heliyon.2023.e14333

**Published:** 2023-03-05

**Authors:** Azadeh Eghbal Manesh, Asghar Dalvandi, Mohammad Zoladl

**Affiliations:** a Department of Nursing, Faculty of Nursing and Midwifery, Tehran Medical Science, Islamic Azad University, Tehran, Iran; b Department of Nursing, Faculty of Nursing and Midwifery, Tehran Medical Science, Islamic Azad University, Tehran, Iran; c Associate Professor of Nursing, Yasuj University of Medical Sciences, Yasuj, Iran

**Keywords:** Stigma, People with schizophrenia, Qualitative research, Family caregivers, And discrimination

## Abstract

**Introduction:**

Schizophrenia is a chronic disabling and the most stigmatizing mental disorder worldwide. The stigma experienced by family caregivers impacts their lives in different ways. This study reports the results of qualitative synthesis to understand the perceptions and experiences of stigma in families of people with schizophrenia spectrum disorders across various socio-cultural contexts.

**Methods:**

An initial comprehensive search was performed in databases like Web of Science, PsycINFO, CINAHL, Scopus, and Ovid-based MEDLINE. By searching, 3560 studies were found, of which 16 articles were included in the present study. A meta-synthesis was done according to the meta-ethnographic approach.

**Result:**

Three themes were generated: perpetuated stigma by general misunderstandings about schizophrenia, mental health inequality contributes to structural stigma, and long-term family caregiving stigmas, attitudes, and coping strategies. These themes indicated the essential experiences of stigma in families of people with schizophrenia, which appeared due to unknown and socio-cultural misconceptions of schizophrenia that led to emotional challenges for family caregivers.

**Conclusion:**

This study addresses stigma-related issues, and coping strategies used almost exclusively by family caregivers. Health policymakers and healthcare professionals working in mental health institutions should consider this data. Substantial steps must be taken to combat stigma, with education initiatives topping the list.

## 1 Introduction

According to the World Health Organization 2020, schizophrenia is a chronic disabling disorder that affects 20 million people worldwide. It impacts how a person thinks, acts, expresses emotions, speaks, and causes hallucinations and delusions [1]. In the general public's view, schizophrenia is associated with undesirable behaviors such as danger and violence [2]. It is one of the most stigmatized mental illnesses [3].

Stigma is a label given to people with personal or physical characteristics that differentiate them from others [4,5]. Self-stigma is the public stigma that people with schizophrenia take on themselves. Affiliative stigma is the stigma people with schizophrenia pass on to their families, friends, and other close relationships [6]. The *social stigma* associated with mental illnesses and the related discrimination makes it problematic for the patients to follow psychological interventions [7,8].

Discrimination manifests as the stigma that impairs many aspects of life, such as work, personal relationships, and education. Because of this, some people with psychiatric disorders may develop irrational prejudices and lose self-confidence, leading to social withdrawal, feeling alone, and shame [9,10].

A family is the smallest social unit, consisting of people linked by marriage or blood relationships and living in the same place [11]. Family members have various responsibilities in caring for a person who has a mental illness—for example, monitoring their psychological and physical problems, transporting them to a hospital or clinic, providing emotional support, financial aid, and so on [10,12]. They also play an essential role in the patient's treatment adherence. Furthermore, family members must tolerate behavioral changes in people with schizophrenia, such as hostility [10,13].

On the other hand, the community believes that the families of people with mental illness are abnormal, influenced by the disorder, and have problematic behaviors [9]. Labels that are attached to people with mental problems can not only stigmatize but also kill them. Because of labeling and stigmatization, families often hide or feel ashamed of people with mental disorders and are embarrassed to take that person to public health centers [14,15]. This worsens the situation of people with schizophrenia and leads to suicidal attempts [16]. Stigma is a complicated experience that impacts all aspects of family life [15]. Many family carers limit their social life not just because they care for someone but also to avoid being stigmatized. In addition to feeling ashamed of their relatives' illnesses, family members also feel guilty, leading to stigma. These feelings often result in a distance from society [17,18].

Yang et al. mentioned, "Stigmas vary across cultures, meanings, practices, and outcomes, even when we see stigma as a powerful and often preferred response to illness, disability, and discord" [19]. For instance, Western countries tend to be individualistic, whereas Asian countries value collectivism. In an individualistic culture, each individual is self-reliant and autonomous. In a collectivist culture, the individual's identity depends on the group and finds meaning in society, leading to intimate relationships. Individual differences are shared in an individualistic culture, while in a collectivist culture, the variety is unusual due to group cohesion. Therefore, collectivism is much more stigmatizing than individualism [20].

So far, various studies have been conducted on the experience and meaning of stigma in families of people with schizophrenia spectrum disorders in different cultures. Therefore, this review presents our detailed meta-synthesis of data from included studies. It provides enriched information about stigma in different cultures that had not been published before and emphasizes the significance of knowing this issue to select suitable anti-stigma interventions. Our study also includes four primary objectives as follow.

1. To investigate experiences of stigma
2. To explain the perception of stigma
3. To illustrate the cultural diversity of stigma
4. Identify strategies for coping with stigma among the family members of people with schizophrenia.

## 2 Materials and methods

This study was a qualitative meta-synthesis, which is a method of interpretating qualitative research reports' findings [21]. The inclusion criteria were [1] studies clarified family caregivers' experiences and perceptions of a relative with schizophrenia in the home setting [2]; qualitative and mixed-method studies used qualitative data as part of their overall data set; and [3] qualitative data that was written in English. Studies were excluded if they were [1] review articles and abstracts [2], meta-synthesis studies on caregiver experiences, or [3] contained disorders other than schizophrenia spectrum disorder.

**Fig. 1:**
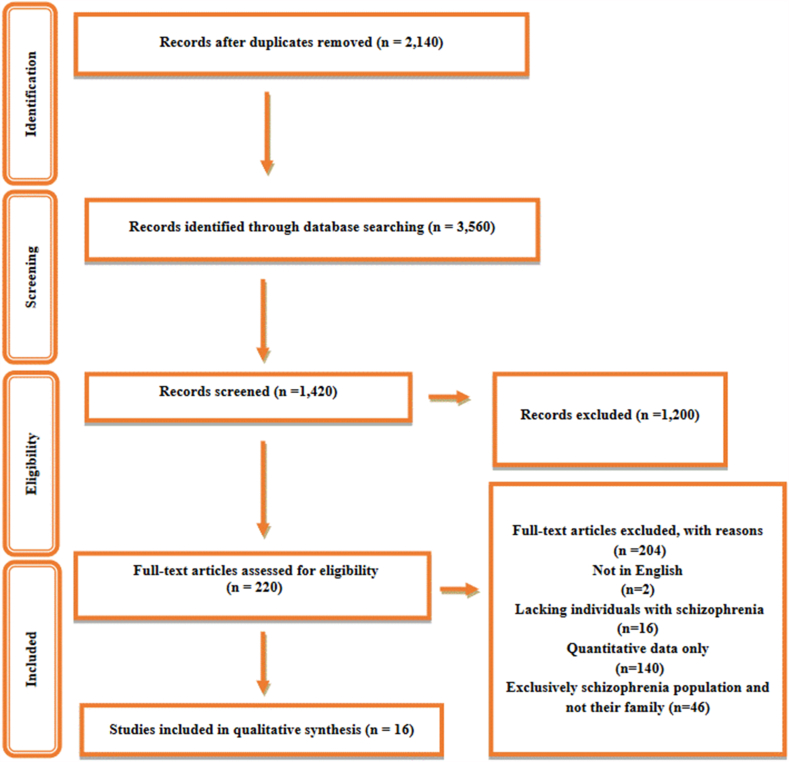
Prisma flowchart – study selection process.

Qualitative research studies were conducted with the help of the university librarian. The researcher started by doing a thorough preliminary search in databases like Web of Science, CINAHL, Scopus, PsycINFO, and Ovid-based MEDLINE. The keywords were [qualitative, OR interview, OR experience, OR phenomenology, OR grounded theory, OR thematic analysis, OR narrative]; and [a family caregiver OR an informal caregiver OR care]; and [a relative, a parent, a family member, a spouse, a husband, a wife, a partner, a brother, a sister, a child, or a sibling]; and [discrimination, OR prejudice, OR discrimination, OR distinction, OR stigma]; and [mental disorder, OR mental illness, OR schizophrenia]. There was no restriction on the publications' date; the search was conducted in June 2021. Reported studies are based on the Preferred Reporting Items for Systematic Reviews and Meta-Analyses (PRISMA) guidelines. The systematic review process is shown in the PRISMA diagram (see Fig. 1).

Endnote received 3,560 studies, of which 2,140 were deleted as duplicates. Then, 1,420 articles were screened for general review, yielding 220 eligible papers. Two hundred and four studies did not meet the inclusion criteria, including 140 quantitative studies; 16 studies lacking individuals with schizophrenia; 46 studies exclusively on schizophrenia populations and not their families; and two articles that were not in English. Three researchers (AE, AD, EM, and MZ) used the inclusion criteria independently, and then they all agreed with a senior researcher (AD) on which papers to include.

We assessed the reporting quality of all included studies using the ten-item Critical Appraisal Skills Program [22]. It contains ten criteria of (a) clarity and appropriateness of purpose and aims; (b) study design; (c) qualitative methodology; (d) sampling method; (e) reflectivity of researchers; (f) data gathering; (g) data analysis; (h) ethics; (i) accuracy of findings; and (j) the importance of research, which is ranked using a three-point scale of no (1 point), I cannot say (2 points), and yes (3 points), resulting in a total overall quality score of 10–30 for each article. Two independent reviewers evaluated the papers and then established a separate second assessment, resulting in agreement on the quality of the documents to minimize any bias. Among the included studies, the majority were of moderate to high quality. Furthermore, several were of lower quality according to the criterion. Table 1 shows the overall distribution of quality ratings for all evaluated criteria. (See Table 1).

**Table 1:** Characteristics and quality scores of included studies.

Source	Country	Methodology	Data Collection	Sample Size	CASP total	Key findings on stigma
Şengün İnan et al. (2021)	Turkey	Content analysis	Semi-Structured Interview	16	25	The stigmatization experience is a complicatedphenomenon. The interviews yielded four themes: the influence of stigmatization on life; dealing with stigma; suggestions for minimizing stigmatization, and the dimensions of stigmatization.
Prokop-Dorner & Flis (2020)	Poland	Content analysis	Observation, expert, and in-depth interviews	11	20	The "zone of stigma" comprises different elements, such as unpleasant conversations, uncertainty, a lack of social cues, healthcare system indifference, psychiatric hesitancy, endangered what's at risk, broken relationships, a stigma triad, and feeling helpless.
Rezayat et al. (2019)	Iran	Content analysis	Unstructured interviews	9	16	The findings include:1. Being ignored socially and humiliated by others, and 2. Coping strategies of discrimination.
Wong et al. (2018)	China	Directed content analysis	Semi-Structured Interview	8	21	The themes consisted of 1. Self-stigma; 2. Feeling humiliated, inadequate, and disappointed; 3. Emotional burden.
Amsalem Doron et al. (2018)	Israel	Grounded theory	Semi-Structured Interview	15	21	There are three basic types of subtle stigma manifestations or microaggressions: 1. Lived experience is irrelevant; 2. People who have psychosis have little chance of remission; 3. Communicating and expressing professional expertise is not required.
Krupchanka et al. (2018)	Czech	Thematic	Semi-Structured Interview	25	25	The main concerns were: 1. A widespread lack of awareness and misunderstandings regarding psychiatric disorders; 2. Structural stigma regardingthe lack of public support networks and the government; and 3. The burden of not being able to live independently and the need for continuous care.
Krupchanka et al. (2017)	Belarus	Thematic	Semi-Structured Interview	20	25	Several themes emerged: (1) mental health care (challenges in accessing mental health experts; obtaining relevant information; few options for hospital treatment; lack of adequate long-term care facilities); (2) work of individuals with schizophrenia; and (3) police interactions.
Paul (2017)	India	Grounded theory	focus-group	14	20	The findings indicated: 1. The effects of stigma on marriage and employment; 2. Gender differences; and 3. Feeling shame and avoidance.
Hyun et al. (2017)	South Korea	Thematic	Semi-Structured Interview	10	23	Six themes emerged: being shamed, feeling ignored, pulling away from friends, getting a mental illness, living as a person who has been accused, and becoming a member of the socially vulnerable.
Krupchanka et al. (2016)	Belarus	Thematic	Semi-Structured Interview	20	23	Stigma is linked to a traumatic event. Guilt, exhaustion, loneliness, worry, and anxietyderive from future uncertainty and grief, which comes from unmet expectations.
Huang et al. (2016)	Taiwan	Thematic	Semi-Structured Interview	15	20	The four main themes were as follows: (a) symptom disturbance is the root of tragedy; (b) an unpleasant view is a lifelong shackle; (c) disorder in a family forces people to work constantly, and (d) not knowing what will happen in the future.
Widodo et al. (2016)	Indonesia	Case descriptive	Free guidance interview	6	12	1. Positive family views result from good socialization, family support, social identity, and surroundings. 2. Self-stigma may be internalized in families and lead to poor self-confidence, changing family attitudes to negative ones.
Mirja Koschorke et al. (2014)	India	Mixed-Method	Semi-Structured Interview	36	25	1. Worries about marriage chances and feelings of being blamed were prevalent. 2. Knowledge was associated with stigma in qualitative but not quantitative 3. Context-specific communications rather than biological information may help in stigma reduction. 4. Caregivers require social and emotional support.
González-Torres et al. (2007)	Basque	Thematic	Focus group	26	18	The findings were separated into three categories: discrimination observed by relatives, discrimination felt by relatives, and discrimination imposed by relatives.
Buizza et al. (2007)	Italy	Grounded theory	Focus group	22	19	Stigma has four components: public view of mental illness; access to social roles adequacy of mental health treatment and stigma internalization.
Schulze & Angermeyer (2003)	Germany	Thematic	Focus group	31	20	The results show that stigma comprises four parts: interactions between people, social roles, institutional discrimination, and how the public thinks about mental disorders.

The primary qualitative data on how people with schizophrenia and their family caregivers experienced and perceived stigma was typed into a Microsoft Word document. Then, each researcher (AE, AD, and MD) did their analysis by reading the document several times, looking for themes, and putting them into groups. The current meta-synthesis followed the seven steps of the Noblet and Harr (1988) synthesis method. This method was used in ethnographic review research, but now it is used in health-related meta-synthesis studies, especially when looking at the experiences of family caregivers of people with chronic illnesses [23,24].

## 3 Result

The results of the present study with language restrictions in inclusion criteria include sixteen qualitative studies published from 2003 to 2021, which demonstrate the difference in stigma perceptions and experiences between family caregivers of schizophrenia patients in European and Asian countries. The researchers elicited all the themes and represented the main findings in Table 1 (See Table 1). According to the overlaps, the research collected data from 286 participants, most of whom were women. However, all studies have not determined the exact number of men and women and their ages. After identifying the field and applying the inclusion criteria, each paper was read and reread to elicit the study themes and methodological information. The researchers compared the themes to see if they were similar. Cross translations occurred, and new themes emerged. Ultimately, three new categories were created and followed as below according to:

[1] perpetuated stigma by general misunderstandings about schizophrenia [2], mental health inequality contributes to structural stigma, and [3] long-term family caregiving stigmas, attitudes, and coping strategies. Each theme is developed from several triangulation-related studies depending on the level at which the stigma occurred.

### 3.1 Perpetuated stigma by general misunderstandings about schizophrenia

There are many misperceptions about people with schizophrenia and their families, which the media exacerbates. For this reason, this theme has two sub-themes as follows.

#### 3.1.1 The media's impact on the prevalence of stigma

There is a lot to learn about physical diseases like AIDS and diabetes. However, there is very little information about psychiatric illness in the community, which might be a good indicator for prioritizing mental and physical diseases in society. People's initial sources of knowledge regarding mental illness are the media, including television programs, newspapers, and the internet [25]. Family members discussed how the media has portrayed people with schizophrenia negatively [[25], [26], [27], [28], [29], [30], [31]]. As one of the family caregivers said:

"Whenever there is a psychosis about violent death, they always mention mental disorders. It seems as though they always give the news from that angle. Generally, the news hits the headlines that way" [27].

#### 3.1.2 The disorder that is often misinterpreted

Family caregivers also pointed out misconceptions and lack of knowledge about schizophrenia in a way that claims people with schizophrenia are considered insane [26,29,30,32].

" … the society has no idea what to imagine under this term (schizophrenia), and if you tell them, they imagine a person who starts killing in a minute or will commit something terrible" [29].

### 3.2 Mental health inequality contributes to structural stigma

Stigma comprises restrictions on mental health services, cultural norms, social structure, and laws that are mentioned below [25].

#### 3.2.1 Stigma regarding mental healthcare services

One of the essential stigmas is the one caused by healthcare professionals. Microaggressions [30,33] and ignoring people with schizophrenia and their families are good examples [29,30,[33], [34], [35]]. Microaggressions are offensive or insulting words or statements that invalidate a person's identity [36]. It is illustrated in one of the family caregivers' quotes below:

"We came here only because we did not know where to go … And what did the doctor tell me? You also act like a lunatic! I felt like I was on fire … " [33].

Also, the family members are sometimes neglected by health professionals and do not receive any information about the disorder. One of the parents pointed this out: "We are treated as objects; we are ignored." Many times, still, as a young person, they talked to me with tongue in cheek. My opinion was not taken into account seriously" [30].

In addition, the family caregivers mentioned the poor quality of mental health services, such as limited insurance coverage, lack of access to medical and non-medical treatment, lack of community-based programs, and shortage of long-term care facilities [25,26,29,30,34]. One of the caregivers stated:

"CT is simply carried out. Nobody would question its necessity and wonder where the money for it should come from" [25].

#### 3.2.2 Stigma regarding cultural norms

According to what Yang et al. indicated, stigma happens in a cultural context; thus, to design anti-stigma interventions, we need to recognize "what matters most or what is at stake" [19]. For example, Confucianism beliefs in East Asia, the importance of "face" in Chinese, the difficulty of getting social roles in Italy, and Hindu philosophy and gender differences in India [26,28,37,38].

#### 3.2.3 Stigma related to social structure

People with schizophrenia encounter several challenges in society after being diagnosed. According to the caregivers, employment and marriage were the most common difficulties they confronted [39]. Stigma has an impact on the employment of people with schizophrenia as well as their family members. Many studies noted the label discrimination in such a way that employers who know that an employee has a mental illness do not hire or pay them wages [9,26,[28], [29], [30],^34^,[38], [39], [40], [41]]. One of the parents stated:

"The employer did not pay a proper salary because he has a mental illness" [28].

Another challenge is the public stigma associated with mental illness; people with schizophrenia often experience neglect, abandonment, and divorce after marriage [18,25,34,37].

"Her husband returned her to me and said, "Come and take back this person with schizophrenia." [39].

In addition to public stigma, mental illness may affect a person's relationships, marriage, and other family members, especially daughters, due to affiliated stigma [18,31,34,42]. As one of the caregivers said:

"If they are getting me married and if the person who is marrying me feels that my mother is like this, I would feel bad (…). They may ill-treat me and dominate me … I fear that" [34].

#### 3.2.4 The stigma associated with laws

People with schizophrenia should be legally protected, so their rights are not violated [37]. According to studies, it is desirable to facilitate crisis services, provide solid legal employment, eliminate much bureaucracy for social care housing, change criminal laws to favor people with mental disorders, and educate police officers on how to contact these people [25,34,39]. One of the family members mentioned this issue below:

"He [the ill family member] does not want to get treatment, denies the illness, and behaves aggressively." A family has a dilemma: whether to go to court and apply for coercive treatment " [30].

### 3.3 Long-term family caregiving stigmas, attitudes, and coping strategies

Some people think schizophrenia disorder is contagious and spreads to everyone, such as afflicted families, leading them to experience affiliative stigma. Therefore, people avoid them [28,29,31].

"It is like they are avoiding a contagious disorder." They do not want to accept us" [31].

Another opinion is that genes transmit this disorder; thus, the family is always blamed [27,30,35,40,43].

"You brought up your son like that; now you need to harvest the crops," the doctor said directly … Furthermore, she did not even prescribe any tranquilizers!" [30].

Moreover, family members encounter numerous difficulties due to their long-term care for people with schizophrenia. Financial and emotional burdens are among the most significant concerns they experience. A financial burden occurs since many people do not have jobs, funds, or government assistance to live independently [25,[28], [29], [30],^32^,^34^,^37^,^39^,^42^]. So, the family caregivers must care for them to the end of their lives. Subsequently, they devote all their time to caring for others, not themselves.

"Before my son's illness onset, I was free of worry and financially sufficient." I did not know this illness was such a devastating one. It made me more distant and made me avoid my relatives. I felt disconnected and disappearing" [28].

Step by step, they experience the emotional burdens more and more. All studies reported that this kind of burden appears with feelings of sadness, guilt, self-blame, embarrassment, helplessness, rejection, avoidance, humiliation, disappointment, pessimism, worthlessness, uncertainty, and powerlessness. Here are some examples of emotional burdens:

"It was the first time I heard the word schizophrenia … I did not know what it meant; I was in such a shock … I was really upset" [33]. "For instance, for the first few years, I was ashamed of having a mentally ill son" [27]. "I was crying and crying. I kept thinking of how to do something with him and his activities" [35]. "Who else may help me? I do everything by myself, and never ask anyone." [34].

Ultimately, the most common ways used by family members to deal with these emotional burdens originating from stigma were avoiding, passively accepting, and internalizing stigma, as well as educating others about the disorder and what causes it. Each of these approaches is explained below, one by one. Most family members utilize avoidance mechanisms to cope with stigma due to public and affiliated stigma. They shun social interactions and hide the person with schizophrenia at home as "life behind closed doors." [28,30,31,34]. Finally, this vicious cycle is constantly repeated and ends in social isolation and emotional pressure.

Another alternative is passive acceptance and internalizing the stigma. This strategy leads to feelings of embarrassment and guilt among family members. So, they will think they are responsible for all of the person's actions [26,31,[33], [34], [35],^38^,^42^].

"It is too late. I shall bear this cross till the end. What else? It is my fate to be with an ill person; my destiny" [34].

In this situation, family caregivers may have irrational concerns or doubts about individuals around them, leading them to perceive that their relatives are looking down on them [28,42]. In other words, they experience anticipatory and internalized stigma [42]. The following is how one of the participants explained it:

"One day, when my sister-in-law told me something related to my son, I was outraged because I thought she was criticizing him. However, I had misunderstood; my sister-in-law had no intention of hurting me" [28].

Despite internalizing stigma by families, Some family caregivers believe other people do not know about this disorder. Consequently they will not be upset by their prejudice and discrimination. Education is one of the essential strategies that have been mentioned in studies. To get rid of stigma in the whole community, education can start with family caregivers and continue through the media [29,31,34,39]. As one of the parents stated:

"We learned about the disorder, too. Moreover, as we learned and accepted it, we found solutions. I tell people around me that if patients take medicines and are under control, there is no need to be afraid" [31].

## 4 Discussion

Though stigma is one of the significant barriers to family caregivers seeking help, active participation in caregiving, and patient adherence to treatment all over the world [44], the findings of our study provided insight into the experiences and perceptions of stigma in family members of people with schizophrenia. Also, these findings make us aware of "what matters most" about stigma in different countries so that anti-stigma strategies can be developed based on them. Three main themes emerged from the data [1]: perpetuated stigma by general misunderstandings about schizophrenia [2]; mental health inequality contributes to structural stigma; and [3] long-term family caregiving stigmas, attitudes, and coping strategies.

According to what was said above, most of the caregivers in the present study were female. In general, female caregivers seemed more committed to caring for individuals with schizophrenia and were often the only ones who remained in relationships with them [42]. Also, according to gender role theory, female caregivers frequently connect deeply with the patients and devote their lives to caring for them [45].

The first theme of our study illustrates the broad misperception about schizophrenia disorder that the media has promoted in most European nations (Table 1). On the other hand, negative attitudes toward schizophrenia are more common in European countries [46]. According to one study, European are more developed than Asian countries regarding resources, social contact, and trust in social networks. Consequently, people accept what the media says, significantly impacting them [47].

Moreover, according to the family caregivers in our study, the second theme is "mental health inequality contributes to structural stigma," which is most prevalent in European countries. One study showed an imbalance between "core health care" and "other care" in different parts of Europe. Other care refers to community mental health nursing, which is ignored in most European countries [48]. Another reason for mental health inequality and negative attitudes may be because, in Asian culture, psychological difficulties are manifested in the form of somatization. Therefore, people seek care for their physical problems rather than therapeutic solutions for their mental illnesses. So, compared to European countries, they rarely complain that mental health services are not good enough [49]. As we previously said, stigma comprises restrictions on cultural norms, social structure, laws, and mental health services [25]. Concerning cultural restriction, in some countries, old traditions and values about people with mental illnesses still lead to the stigma titled "stigma regarding cultural norms." For instance, Confucianism is a philosophical belief system in South Asian countries, especially China and South Korea. Moral principles in interpersonal interactions and society are frequently classified as Confucianism, which focuses on cohesion and harmonic social relationships based on cultural norms. Since some people with schizophrenia cannot act like others in society, according to Confucian philosophy, they will be labeled as abnormal and stigmatized [50]. Based on this philosophy, individual needs should be sacrificed for the welfare of the collective. One study discussed Confucianism as the most prevalent philosophical and religious belief among Chinese people, which can result in self-stigma and affiliated stigma [41].

In addition, in collective cultures, people's desire to keep their social image, ideals, and status as impacted by their performance and specialized social positions has been frequently noted [51]. One of the Chinese proverbs about the importance of the face says: "A person needs face just as a tree needs bark " [52]. Face concern is associated with feelings of shame and stigma internalization. One similar study found that people with mental disorders and their families in some Asian communities, such as Chinese and Korean, do not talk about their illnesses to keep their faces, which can delay treatment [53]. While, In their study, Krupchanka et al. indicated that the Czech Republic's individualist culture advocated productivity and independence. This study also mentioned that sometimes people with schizophrenia could not even take care of themselves, so in the individualistic culture, they will be labeled as "parasites, "leading to stigma [29]. Regarding this research, one study highlighted the role of social skills in reducing self-stigmatization. In Italian culture, having a social role is very important because it makes people with mental illness feel helpful, and as a result, they rarely internalize their stigma [54]. Similar to Italian culture, In India, Hindu philosophy says that doing one's responsibility is a crucial part of living morally and that following Dharma's rules will make the mind more refined, leading to Moksha (liberation). One study, the same as the current one, found that Indians think working and getting married are essential and that if a person with a mental disorder cannot do them, it leads to stigma [55].

It is worth noting that most of the countries in our synthetic research experienced stigma in employment and marriage. One study showed that low and middle-income countries experienced social stigma because of their socioeconomic status. For example, in India and Iran, being productive for men and women is very important [56]. Grover et al. (2017) reported gender differences in stigma in India, similar to this study. In this regard, women must serve the family, and marriage is essential for them, just like men's employment [57]. In harmony with our research, the findings of another study regarding developing countries in Asia demonstrated that mental illness results in social disadvantages like divorce and marital separation [58]. In addition, another study conducted in European countries found that employment is one of the predictors of decreased self-stigmatization and empowerment [59].

Sometimes stigma arises for people with schizophrenia and their families when there are no rules to deal with them. For this reason, in our study, laws are established in some societies to cope with this stigma. In line with this study, the results of another study showed that putting policies and laws into place in different parts of the country leads to less stigmatization [60]. Finally, in the continuation of this research, other ways of dealing with stigma are mentioned below. The last theme discussed is "long-term family caregiving stigmas, attitudes, and coping strategies." According to what the family caregivers have indicated, most of them feel a high emotional burden, which prompts them to use coping mechanisms, which may be adaptive or maladaptive, depending on the situation. This is in line with the findings of the research conducted by Javed et al., which revealed that most family carers worldwide experience a burden. In addition, they stated that family caregivers in low and middle-income countries suffered a more significant burden due to the limited accessibility of resources [56]. Different strategies to cope with stigma depend on the underlying local culture. As previously said, stigma has various reasons in different nations, which may require different strategies for dealing with it. In the current study, the most common strategies for family members to cope with stigma were avoiding, passively accepting, internalizing stigma, and educating people about the disorder and what creates it. Keeping face is very important in South Asian countries such as Korea and China, according to what was described above. Because of this, family caregivers in these countries hide the disorders of their loved ones. They also avoid relationships with others out of fear of being affiliated and self-stigma [41].

Moreover, people tend to be self-reliant in an individualistic culture, so deviation is not as crucial as in collectivism. For instance, Taiwan is individualist, and China is more collectivist than Taiwan [43]. In a collective culture, people feel more embarrassed and ashamed of caring for someone with a mental disorder. Therefore, their desired coping strategy is to avoid other people, hide the person with a mental disorder, and tolerate more emotional burdens. A deviation is accepted in individualist Western countries like Germany, Italy, and Spain. People do not have to hide their loved ones with mental disorders; instead, they do not show avoidance attitudes toward them. In individualistic countries, people expressed a preference for participating in rehabilitation activities [7,8,20,29,39,42,49,56,61].

Sometimes religion significantly impacts individuals' cultures, like Hindus in India, since the followers of Hinduism believe that we came to this world to fulfill our responsibilities [42]. Whereas in our study, the influence of religion on culture in Iranian people is not dominant, and holding ceremonies leads to prying. In contrast, another study in Iran declared that these ceremonies with the prayers of Muslims are approaches that the Iranians use to cope with stigma [62].

In summary, this discussion emphasizes the necessity and importance of recognizing stigma in the socio-cultural context of Asian and European countries. Based on this, mental healthcare professionals should select appropriate anti-stigma interventions and spread them at the community level.

## 5 Limitations

The present study had a small sample size, offset by a reasonable interpretation. Another search criterion that may limit transferability is the English language. Therefore, it is preferable to look for publications in different languages in future studies. It is important to compare our findings to those of other psychiatric disorders to identify the key components affecting stigma for family caregivers of people with schizophrenia.

## 6 Conclusion

This study addresses stigma-related issues, and coping strategies used almost exclusively by family caregivers. Health policymakers and healthcare professionals working in mental health institutions should consider this data. Essential steps should be taken to combat stigma, including the following: developing a health education program, which should be promoted to the people of the community; involving family caregivers in the decision-making and policymaking processes; the creation of a tool to improve the accessibility of information and facts about the disorder, such as advertisements, media coverage, TV broadcasts, and so on; the resocialization and independent living of people with schizophrenia; improving mental health services by developing access to care in communities; and increasing family support services.

## 7 Implications for clinical practice and research

This study indicates that the stigma experienced by family caregivers impacts their lives differently. To prevent stigma at the community level, we must inform people about schizophrenia and how it occurs. Increasing media use can also be an operational approach. To combat stigma at the governmental level, it is required to do action research in each country to know the factors related to stigma based on their culture. Additionally, health professionals should use education programs according to their socio-cultural context to support family caregivers in dealing with stigma.

Further research is needed to investigate the effect of stigma on family caregivers' seeking behavior. A future meta-synthesis study is recommended without language restriction. At the familial level, the family caregivers of people with schizophrenia should be assessed physically and mentally, and psychiatric consultation should be done if they need it. Their coping strategies also need to be looked at to see whether they are adaptive or maladaptive.

## Authors’ contributions

All planned the study, interpreted the results, and wrote the manuscript. All authors read and approved the final manuscript.

## Funding statement

No funding was used in this study.

## Disclosure

None of the authors has any conflict of interest with the information presented in the manuscript.
